# Association between unhealthy food consumption and mental health outcomes in children and adolescents: an umbrella review

**DOI:** 10.1186/s13034-026-01079-4

**Published:** 2026-04-02

**Authors:** Clayton Ulm, Frantasia Hill, Sarah D. Mills, Carolyn Chelius, Eric M. Hecht

**Affiliations:** 1 Department of Health Behavior, Gillings School of Global Public Health, University of North Carolina at Chapel Hill, 170 Rosenau Hall, CB #7440, Chapel Hill, NC 27599-7440 USA; 2 Institute of Etiological Research, Boca Raton, FL USA; 3 Lineberger Comprehensive Cancer Center, University of North Carolina, Chapel Hill, USA; 4 Charles E. Schmidt College of Medicine, Florida Atlantic University, Boca Raton, FL USA; 5 Department of Public Health Sciences, University of Miami Miller School of Medicine, Miami, FL USA

**Keywords:** Unhealthy food, Ultra-processed foods, Mental health, Children, Adolescents, Umbrella review

## Abstract

**Background:**

The consumption of unhealthy food has increased substantially among children and adolescents across the globe. This umbrella review aims to synthesize meta-analyses that assess unhealthy food consumption and mental health outcomes in children and adolescents.

**Methods:**

The protocol was pre-registered with PROSPERO (CRD42023475965). We searched PubMed/MEDLINE, Scopus, Embase, and CINAHL from inception through January 2025 for meta-analyses that estimated associations between unhealthy food exposures and mental health in children and adolescents (< 19 years). Exposures included ultra-processed food (UPF), junk food, World Health Organization unhealthy food indicators (e.g., sweets), and Western dietary pattern. Extracted meta-analyses were replicated, re-evaluated for credibility, and assessed for methodological quality.

**Results:**

With a sum total sample size of 2,526,232, our search yielded 15 pooled estimates. Convincing evidence (Class I) linked higher UPF consumption to short sleep duration (odds ratio = 1.30, 95% CI [1.26, 1.33]) and insomnia (odds ratio = 1.47, 95% CI [1.37, 1.57]). Highly suggestive evidence (Class II) related higher UPF consumption to poor sleep quality (odds ratio = 1.53, 95% CI [1.46, 1.60]), and common mental disorders (odds ratio = 1.53, 95% CI [1.44, 1.63]), and higher junk food consumption to depression (odds ratio = 1.62, 95% CI [1.39, 1.90]). Suggestive (Class III) or weak (Class IV) evidence related junk food to anxiety and stress, and junk food, sweets, and Western dietary patterns to attention-deficit/hyperactivity disorder. Associations with sleep dissatisfaction and happiness were not statistically significant.

**Conclusion:**

Findings suggest that children and adolescents who eat more unhealthy food tend to have poorer mental health. Nutrition-based interventions should prioritize children and adolescents since this population may be particularly susceptible to diet-related poor mental health. Further causal-inference studies should evaluate whether lowering consumption improves youth mental health.

**Supplementary Information:**

The online version contains supplementary material available at 10.1186/s13034-026-01079-4.

## Introduction

In recent decades, the consumption of unhealthy food has increased substantially among children and adolescents across the globe [1, 2]. Unhealthy foods provide little nutritional value relative to their caloric content and have often undergone substantial processing before consumption. Unhealthy foods are typically high in refined carbohydrates, added sugar, saturated fat, sodium, and low in fiber, protein, and most vitamins and minerals [3, 4]. Unhealthy foods can be defined in many ways. Some operationalizations are based on nutrient profiles (e.g., junk foods, sweets), industrial processing (e.g., ultra-processed foods [UPFs]), or cumulative dietary pattern (e.g., Western Diet). UPFs, a widely used definition of unhealthy foods, contain numerous additives, including fillers, emulsifiers, flavorings, and colorings [2]. In the United States, the proportion of energy intake from UPFs among adolescents has increased to nearly 70% as of 2018 [5].

The incidence and prevalence of poor mental health among United States (US) children and adolescents has also been growing and was recently declared a crisis by the US Surgeon General [6]. Between 2016 and 2022, diagnoses of anxiety or depression among US children aged 3–17 increased from 7% to 11% and from 3% to 5%, respectively [7]. From 2013 to 2023, the proportion of US high schoolers who had at least 8 h of sleep on an average school night fell from 32% to 23% [8]. During the same period, there was an increase in the percentage of US high school students who reported seriously considering suicide (17% to 20%) and attempting suicide (8% to 9%) [8]. Similarly, the percentage of US 3–17 year olds diagnosed with attention-deficit/hyperactivity disorder (ADHD) has increased from 6% (1997–1998) to 11% (2022) [9, 10].

Mental health is, in part, dependent on the health of many intricately connected organ systems [11–13]. Research finds that an unhealthy diet may harm mental health by influencing the delicate heart-brain, liver-brain, gut-brain, and other body-brain axes [12, 14]. In addition, childhood and adolescence are neurodevelopmental periods during which the brain is particularly vulnerable to the adverse effects of a poor diet and obesity [15, 16]. Although several reviews suggest unhealthy foods are associated with worse mental health [17–19], a comprehensive systematic review of the effect sizes associated with various unhealthy foods and mental health outcomes is lacking. Therefore, to synthesize the literature, we conducted an umbrella review of meta-analyses to determine which childhood and adolescent mental health outcomes are related to the consumption of unhealthy food and to assess the strength of these associations.

## Methods

### Search strategy

We systematically searched the literature following Preferred Reporting Items for Systematic Reviews and Meta-Analyses (PRISMA) guidelines listed in Supplementary Table A [20]. The study protocol was pre-registered with the International Prospective Register of Systematic Reviews (PROSPERO; ID: CRD42023475965), a registry for a priori reporting of systematic review protocols. We defined the research question using the PICOS criteria shown in Supplementary Table B. Our primary outcomes included physiological health and obesity outcomes, but we present only mental health outcomes in this review. We searched PubMed/MEDLINE, Scopus, Embase, and CINAHL from inception to January 2025 for meta-analyses estimating the mental health effects of unhealthy food exposures in children or adolescents. To be included, studies had to: (1) focus on infants, children, or adolescents (< 19 years), (2) be a systematic review with a meta-analysis containing an odds ratio (OR), risk ratio (RR), or hazard ratio (HR), and (3) be written in English. To capture reviews on an array of unhealthy food exposures, we included meta-analysis studies that assessed the following four categories of unhealthy food consumption: (1) NOVA-classified UPFs, which are defined as industrially processed food products that use culinary preparation techniques and substances not typically found in households [21]; (2) World Health Organization (WHO) published indicators of unhealthy food consumption, defined as foods high in free sugars, saturated fats, salt, and low in nutrient density (e.g., sweets, candies, chocolate, cakes, and cookies) [22]; (3) junk food, which are foods high in added sugars, fat, and calories with little to no nutritional value and minimal essential nutrients [23]; and (4) Western dietary pattern, which is typically high in refined grains, soft drinks, and processed meats, but also minimally processed products like high-fat dairy products and red meat [24]. There is significant overlap in these categories (e.g., junk food is almost exclusively ultra-processed), but they are also distinct (e.g., not all UPFs are junk foods). The search terms are listed in Supplementary Table C.

### Screening and selection

Duplicate references were removed before screening. Two independent reviewers (CU, FH) screened titles and abstracts based on the inclusion/exclusion criteria. Next, reviews of full articles were performed. One (CU) reviewer screened all articles, and a second reviewer (FH) screened approximately half of the titles/abstracts and full-text articles in duplicate. Inter-rater reliability between the reviewers was substantial for titles/abstracts (Cohen’s Kappa = 0.78, 96.7% agreement) and full-text (Cohen’s Kappa = 0.73, 92.3% agreement). Conflicts between reviewers were resolved by a third reviewer (SM). Covidence, a web-based screening and data extraction software, supported the systematic review process [25].

### Data extraction process

We extracted information from primary studies included in each meta-analysis that met our inclusion criteria. When available, we prioritized extracting estimates from adjusted models. From primary studies, we extracted OR, 95% confidence intervals (CIs), study designs, sample sizes, authors, publication years, exposures, and outcomes. We also collected information from the pooled effects reported in each meta-analysis. We extracted the number of effects combined (*k*), the sample size of the pooled effect (*N*), whether the number of cases was > 1,000, and whether the largest study included in the pooled effect was statistically significant. Two reviewers (CU, FH) extracted all data in duplicate and resolved disagreements through discussion.

### Data analysis

All included meta-analyses were replicated using metafor and meta packages in R to standardize pooling methods. Random-effects restricted maximum likelihood models were used to pool effect sizes when *k* > 3; otherwise, fixed-effects models were used to avoid the unstable estimation of between-study variance when the number of primary studies is very small [26]. Publication bias was assessed using Egger’s and Begg’s tests [27, 28]. A test of excess significance bias was used to detect overrepresentation of significant results [29]. Sensitivity analyses were performed to address primary study overlap by excluding duplicate primary studies appearing in more than one meta-analysis. For meta-analyses with substantial heterogeneity (I^2^ > 50%), we used a sequential exclusion algorithm approach to iteratively exclude one or more studies until the I^2^ fell below 50% [30].

### Credibility of evidence assessment

We evaluated the strength of reported associations using a framework based on established standards (see Supplementary Table D). These criteria use statistical parameters to assess the reliability of pooled effect estimates [31]. We classified evidence into five categories: convincing (Class I), highly suggestive (Class II), suggestive (Class III), weak (Class IV), and non-significant (Class ns). These categories indicate the robustness and reliability of the association, but not causality. Convincing (Class I) estimates are unlikely to be influenced by future similarly designed studies, while lower classes may change with additional data.

### Methodological quality assessment

We used the second iteration of A Measurement Tool to Assess Systematic Reviews (AMSTAR 2) tool to assess the methodological quality of each systematic review [32]. AMSTAR 2 evaluates 16 domains, including protocol registration, literature search, study selection and data extraction, and bias assessment [32]. Two reviewers (CU, FH) independently assessed each review, resolving discrepancies through discussion. Following AMSTAR 2 guidelines, we did not create a composite score but evaluated each domain, emphasizing critical weaknesses [32]. Ratings ranged from high (no or one non-critical weakness) to critically low (more than one critical weakness).

## Results

**Fig. 1:**
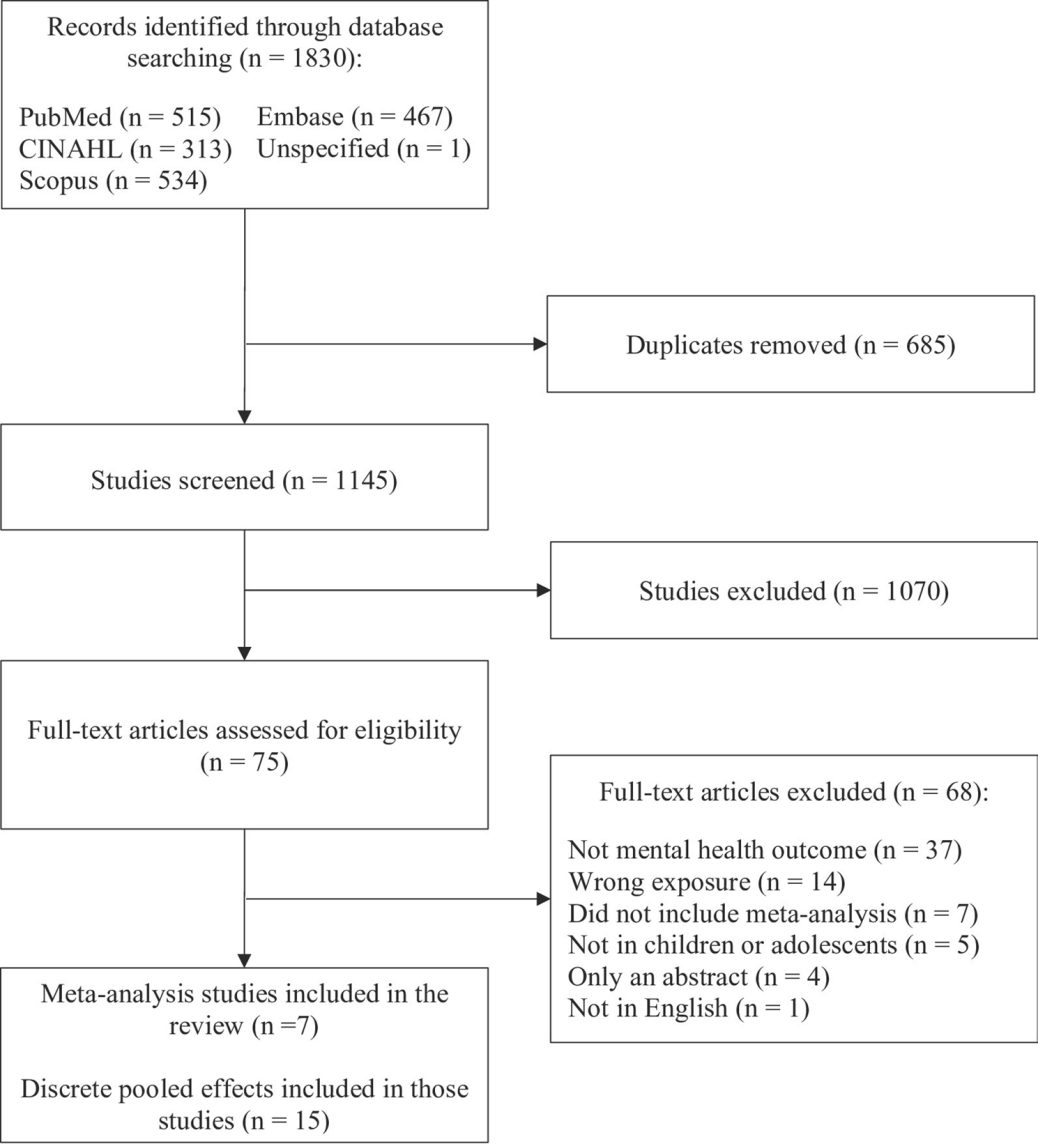
Flow chart of the screening process

**Fig. 2:**
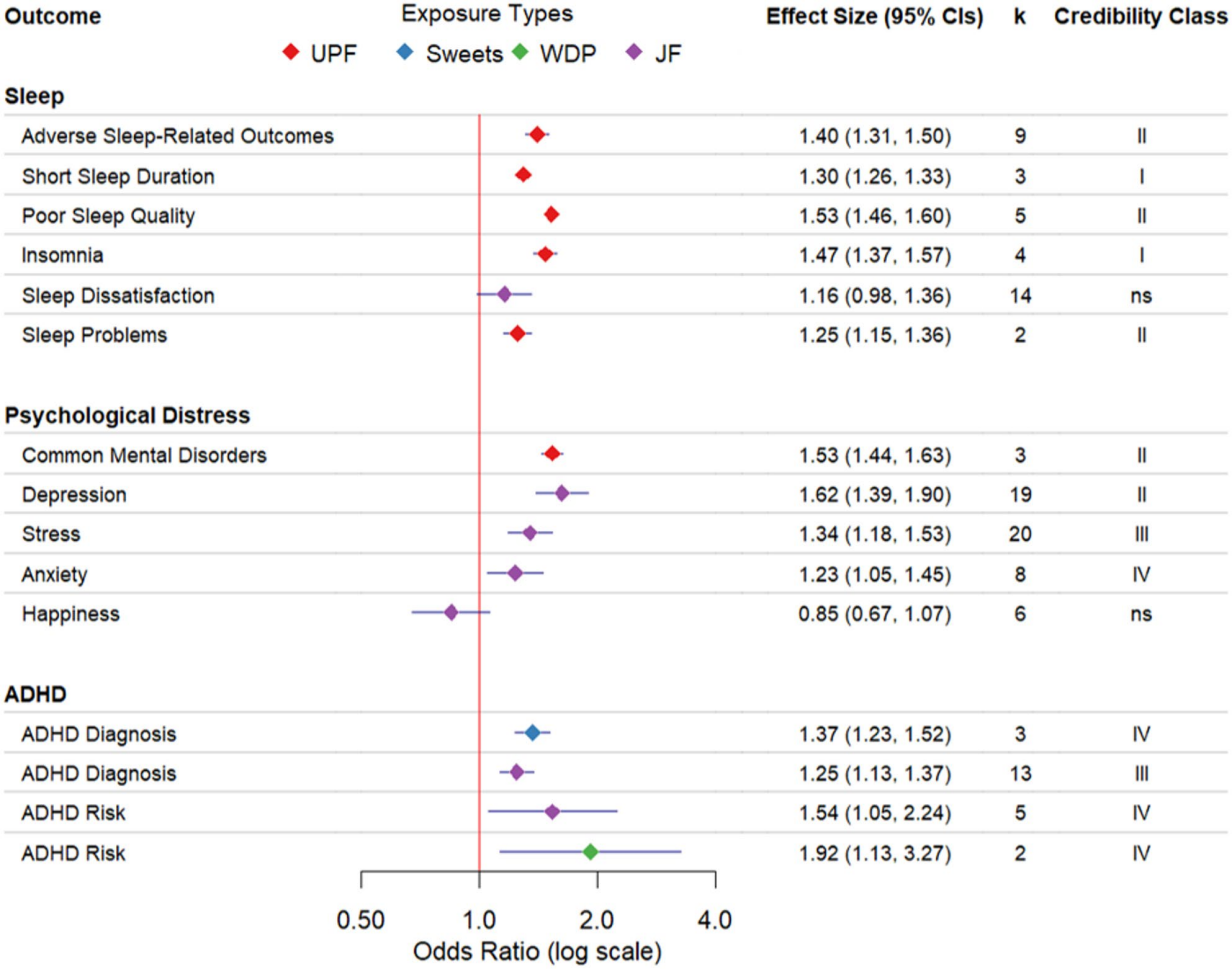
Forest plot of unhealthy food consumption and mental health outcomes in children and adolescents. The plot displays pooled odds ratios and 95% confidence intervals (CIs) color-coded by exposure type: red - ultra-processed foods (UPF), blue - sweets, green - Western dietary pattern (WDP), and purple - junk food (JF). K=number of unique effect estimates included in each meta-analysis. Credibility of evidence assessments: Class I=Convincing, II: Highly Suggestive; III: Suggestive; IV: Weak; and ns: Non-significant; ADHD: Attention-Deficit/Hyperactivity Disorder

Our search yielded 1,830 articles. After removing 685 duplicates, 1,145 titles and abstracts were screened. Seventy-five full-text articles were reviewed for eligibility, of which 68 were excluded for reasons identified in Fig. 1. Of the meta-analysis studies evaluated, seven were included in this review (publication years ranged from 2020 to 2024). Fifteen pooled effect estimates quantifying the relationship between consumption of unhealthy food and mental health outcomes were extracted. The sum total sample size across all pooled effect estimates was 2,526,232 individuals (individual meta-analysis sample sizes ranged from *n* = 1,641 to *n* = 488,441). Exposures included high consumption of UPF (*n* = 6) [18, 33–35], junk food (*n* = 7) [17, 19, 36], sweets and candy (*n* = 1) [36], and Western dietary pattern (*n* = 1) [19]. We grouped our outcomes into three mental health domains: (1) sleep (*n* = 6), which includes sleep duration, quality, and perceived dissatisfaction, and insomnia [17, 18, 33, 34], (2) psychological distress (*n* = 5), including depression and anxiety, and other specific measures of distress like stress and happiness [17, 35], and (3) ADHD (*n* = 4), measures used to diagnose ADHD or assess symptoms of hyperactivity [19, 36]. Each pooled effect is summarized below, with statistical parameters detailed in Table 1 and visualized in Fig. 2.

**Table 1:** Characteristics of included meta-analyses

Exposure	Exposure Measure	Outcome	Effect Size	95% CIs	I² (%)	k	*N*	*n* Cases > 1,000	*P*-value	LS	SSE	ESB	CE	Study designs
**Sleep**														
UPF [18]	Quantiles/FFQ	Adverse Sleep-Related Outcomes	1.40	(1.31, 1.50)	92.8	9	488,441	Yes	1.18E-21	sig.	ns.	ns.	II	Cross-sectional: 9
UPF [18]	Quantiles/FFQ	Short Sleep Duration	1.30	(1.26, 1.33)	37.3	3	308,837	Yes	6.56E-85	sig.	ns.	ns.	I	Cross-sectional: 3
UPF [18]	Quantiles/FFQ	Poor Sleep Quality	1.53	(1.46, 1.60)	56.9	5	291,537	Yes	1.29E-72	sig.	ns.	ns.	II	Cross-sectional: 5
UPF [33]	Quantiles/FFQ	Insomnia	1.47	(1.37, 1.57)	0.0	4	114,998	Yes	3.82E-28	sig.	ns.	ns.	I	Cross-sectional: 4
JF [17]	FFQ	Sleep Dissatisfaction	1.16	(0.98, 1.36)	99.7	14	254,187	Yes	7.64E-02	sig.	ns.	ns.	ns	Cross-sectional: 5
UPF [34]	FFQ	Sleep Problems	1.25	(1.15, 1.36)	90.7	2	3,375	Yes	2.17E-07	sig.	sig.	ns.	II	Cross-sectional: 2
**Psychological Distress**														
UPF [35]	Quantiles/FFQ	Common Mental Disorders	1.53	(1.44, 1.63)	51.9	3	170,218	Yes	2.69E-43	sig.	ns.	ns.	II	Cross-sectional: 2
JF [17]	FFQ	Depression	1.62	(1.39, 1.90)	99.7	19	219,482	Yes	9.05E-10	sig.	ns.	ns.	II	Cohort: 1 Cross-sectional: 5 Case-control: 1
JF [17]	FFQ	Stress	1.34	(1.18, 1.53)	99.6	20	395,860	Yes	7.81E-06	sig.	ns.	ns.	III	Cross-sectional: 5
JF [17]	FFQ	Anxiety	1.23	(1.05, 1.45)	73.3	8	13,886	Yes	1.09E-02	sig.	ns.	ns.	IV	Cross-sectional: 2
JF [17]	FFQ	Happiness	0.85	(0.67, 1.07)	97.3	6	196,283	Yes	1.55E-01	sig.	ns.	sig.	ns	Cross-sectional: 3
**ADHD**														
Sweets [36]	Quantiles/FFQ	ADHD Diagnosis	1.37	(1.23, 1.52)	0.0	3	1,641	No	2.64E-09	sig.	ns.	ns.	IV	Cohort: 1 Cross-sectional: 1 Case-control: 1
JF [36]	Quantiles/FFQ	ADHD Diagnosis	1.25	(1.13, 1.37)	55.1	13	58,296	Yes	8.61E-06	sig.	ns.	ns.	III	Cohort: 2 Cross-sectional: 5 Case-control: 2
JF [19]	Quantiles/FFQ	ADHD Risk	1.54	(1.05, 2.24)	53.4	5	5,948	Yes	2.62E-02	sig.	sig.	ns.	IV	Cohort: 1 Cross-sectional: 2 Case-control: 2
WDP [19]	Quantiles of adherence score	ADHD Risk	1.92	(1.13, 3.27)	0.0	2	3,243	No	1.61E-02	sig.	ns.	ns.	IV	Cohort: 1 Cross-sectional: 1

WDP: Western Dietary Pattern; JF: Junk Food; UPF: Ultra-Processed Food; OR: Odds Ratio; CI: Confidence Interval; LS: largest study statistical significance; SSE: Small study effects (publication bias); ESB: Excess significance bias; CE: credibility class of evidence; na: Not Applicable; sig.: Significant; ns: Not Significant; ADHD: Attention-deficit/hyperactivity disorder, FFQ: Food frequency questionnaire. Quantiles are the division of exposure distribution based on % of total energy or weight (e.g., top quartile vs. bottom quartile). Adherence score was generated from a principal component analysis to measure how closely diet matched a Western dietary pattern

### Sleep

Extracted pooled effects consistently indicated that higher consumption of unhealthy foods was associated with increased risk of adverse sleep-related outcomes (see Table 1; Fig. 2). In one analysis, higher UPF consumption was associated with worse sleep-related outcomes, including shortened sleep duration (i.e., < 9 h/day for 6–12 years, < 8 h/day for 13–17 years) and poorer sleep quality (i.e., sleep-quality scales or self-reported difficulty falling asleep/unsatisfactory sleep) [18]. In another analysis, higher UPF consumption was associated with a greater likelihood of insomnia (i.e., assessed with validated insomnia scales or diagnostic criteria) [33]. In addition, a smaller review associated UPF consumption with sleep problems (i.e., sleep quality index, daytime sleepiness scale) [34]. Although a pooled estimate indicated that greater consumption of junk food was associated with increased subjective sleep dissatisfaction, our replication analysis did not reach statistical significance (*p* = 0.08) [17].

### Psychological distress

Extracted associations indicated that unhealthy food consumption was associated with increased risk of psychological distress (see Table 1; Fig. 2). UPF consumption was linked to an increased risk of common mental disorders (i.e., item measures of depression, anxiety, and anxiety-induced sleep disturbances) [35]. Another study in children and adolescents aged 4–18 years found associations between higher junk food consumption and increased risk of depression (i.e., diagnosis or antidepressant prescription, scales and single measures of depression), stress (i.e., single item perceived stress, stress scales, posttraumatic stress symptoms), anxiety (i.e., scales of anxiety), and lowered happiness (i.e., questionnaires of well-being and happiness), although happiness was not statistically significant in our replication analysis (*p* = 0.16) [17].

### Attention-deficit/hyperactivity disorder

Pooled estimates related higher unhealthy food consumption to increased risk of ADHD. A review found that higher consumption of both junk food and Western dietary patterns was associated with an increased risk of ADHD (i.e., questionnaires for ADHD symptoms or subscales for hyperactivity symptoms) [19]. In a separate review, junk food and sweets/candies consumption was associated with increased odds of ADHD diagnoses (i.e., scales, checklists to diagnose ADHD or hyperactivity) [36].

### Methodological quality

Our AMSTAR 2 assessment yielded predominantly low methodological quality ratings: three studies received ‘critically low,’ three received ‘low,’ and only one received ‘high-quality’ (see Supplementary Table E). Common critical weaknesses included a lack of protocol pre-registering (57.1%; AMSTAR 2 Question 2), not accounting for the risk of bias when discussing results (28.6%; AMSTAR 2 Question 13), and failing to investigate and discuss the impacts of publication bias (42.9%; AMSTAR 2 Question 15).

Sensitivity Analyses.

Most primary study estimates were used only once (*k* = 92/103, 89.3%) (see Supplementary Figure A). However, there was some overlap in primary study effects. Only one primary study was used in two separate systematic reviews. In addition, two reviews included overlapping primary studies across meta-analyses conducted in the same systematic review. To address overlap in primary studies, we conducted a sensitivity analysis removing duplicate primary studies (see Supplementary Figure B). Substantial heterogeneity (I^2^ > 50%) was observed for 11 of 15 pooled effects. After excluding studies to reduce I^2^ to below 50%, changes were subtle in most cases (see Supplementary Figure C). However, the adverse associations between junk food consumption and happiness (OR = 0.68, 95% CI [0.59–0.78]) and sleep dissatisfaction (OR = 1.40, 95% CI [1.29–1.51]), which were not significant in the primary replication, became significant.

## Discussion

We found 13 significant associations between unhealthy food consumption and adverse mental health outcomes across three domains: sleep, psychological distress, and ADHD. Convincing (Class I) evidence was found for associations between unhealthy food consumption and increased risk for short sleep duration (UPF) and insomnia (UPF). Highly suggestive (Class II) evidence was found for increased depression (junk food), adverse sleep-related outcomes (UPF), poor sleep quality (UPF), sleep problems (UPF), and common mental disorders (UPF). Evidence linking unhealthy food consumption to ADHD was of lower credibility. Our findings indicate that diet is closely associated with several poor mental health conditions among children and adolescents.

### Strengths and limitations

Our study has several strengths. We pre-registered our study and used rigorous methods, including duplicate study selection and credibility and quality assessments. We adhered to PRISMA guidelines for meta-analysis reporting and provided a transparent account of our methods. We included studies that used multiple definitions of unhealthy foods (e.g., Western Diet, sweets, junk food), thereby allowing us to capture studies that measured similar dietary patterns. In addition, meta-analyses included in our study are globally relevant because they include several studies from high-income countries (e.g., the US; [37]), middle-income countries (e.g., Brazil; [37]), and some studies in low- and lower-middle-income countries (e.g., Bangladesh; [36]).

This study also has limitations. The majority of primary studies used to pool evidence in the meta-analyses were cross-sectional, which limits inferences on temporality and causality. For example, the association between UPF consumption and insomnia and shorter sleep may be subject to reverse causality and bidirectionality, as documented in the literature [37]. We did not exclude meta-analyses that were secondary outcomes of a systematic review. For example, Lane et al. (2022) conducted their primary meta-analysis in the general population but provided specific pooled estimates for adolescents as a part of a subgroup analysis [35]. The broad range of unhealthy food categories with varying definitions also posed a limitation. While this was a strength of our study, exposure variation may have introduced inconsistencies in effect estimates. Most meta-analyses showed high heterogeneity (I^2^ > 50%), indicating variability across primary study effect estimates. Sensitivity analyses produced more consistent results. However, additional subgroup analyses accounting for differences in methods, study designs, populations, inclusion criteria, and exposure measurement may explain the heterogeneity.

The low methodological quality of most meta-analyses in our study was also a limitation. Many studies lacked documentation of established methods prior to review, which can increase the risk of selective reporting bias. Another issue was inadequate investigation of publication bias, which refers to the disproportionate publication of studies with significant results in the expected direction (e.g., higher unhealthy food consumption associated with adverse mental health outcomes). Additionally, many studies failed to consider risk of bias, such as using the Newcastle-Ottawa Scale, when interpreting findings. Ignoring potential bias risks overestimating effects and drawing misleading conclusions. Studies with higher methodological rigor, such as those confirming the association between increased UPF intake and insomnia, should carry greater weight. More rigorous methods are needed to strengthen evidence for relationships between UPF consumption and stress, anxiety, happiness, and ADHD.

### Future research

Additional research is needed to clarify the association between UPF consumption and mental health outcomes in children and adolescents. Large prospective cohort studies with repeated dietary assessments over time could establish temporality. Ethically conducted interventions or clinical trials that reduce UPF intake could strengthen causal claims. Currently, no meta-analyses have examined associations between UPF consumption and eating disorders or food addiction. Lane et al. (2022) presented narrative evidence linking UPF consumption to food addiction in children and to eating disorders in a mixed sample of adolescents and adults [35]. However, they were unable to perform a meta-analysis for these outcomes due to the limited number of available primary studies [35]. Nonetheless, our findings align with eating disorder risk pathways. In prospective cohorts, sleep disturbance, insomnia symptoms, and shorter sleep predicted later binge eating in early adolescence [38], and childhood anxiety symptoms predicted eating disorder symptoms and diagnoses in adolescence [39]. These pathways suggest that unhealthy food consumption could be a risk factor for later development of eating disorders among youth and warrant further investigation of this association.

Studies analyzing the underlying biological mechanisms by which UPF consumption affects mental health could enhance our understanding and further support a causal relationship. The health of multiple organ systems is related to brain health and mental health [11–13]. For example, low fruit and vegetable and high processed food intake precedes poorer musculoskeletal, immune, metabolic, and liver health, which, in turn, predicts higher depression, anxiety, and neuroticism [12]. Therefore, this research in adults suggests that an unhealthy diet may harm mental health through these pathways [12, 14]. However, mechanistic research on children and adolescents is less well-studied.

### Implications and policy relevance

Nutrition-based interventions should prioritize protecting children and adolescents since this population may be particularly vulnerable to the mental health harms of a poor diet and obesity [15, 16]. Several countries, particularly in Latin America, have implemented policies to lower the consumption of ultra-processed and other unhealthy foods [40]. These policies include taxing these products, requiring front-of-package health warning labels, banning the marketing of UPFs to children, and limiting their availability in schools [40]. Evidence from real-world studies in Mexico, Chile, and California suggests that these policies reduce purchases of specific unhealthy foods (e.g., sugar-sweetened beverages and packaged foods) and may lower youth obesity [41–43]. The US has not yet implemented national policies such as taxes, warning labels, or marketing restrictions. However, over the past 20 years, an increasing number of federal and state initiatives have aimed to reduce UPF consumption among children and adolescents, including regulations governing UPF availability in childcare facilities and schools [44]. A review of US federal and state policy documents since 1983 identified 25 policy actions mentioning UPFs, of which 22 were proposed or passed between 2011 and 2022 [44]. These efforts suggest an increasing public awareness and policy focus on addressing the potential harm that UPFs pose to youth [44]. The evidence synthesized in this review may inform evaluations of policies aimed at reducing UPF consumption to assess their potential impact on youth mental health.

## Conclusion

Findings from this umbrella review suggest that children and adolescents who consume more unhealthy food also tend to have poorer mental health. This includes poorer quality or less sleep, more psychological distress, and a greater risk for ADHD, with especially consistent evidence for insomnia and short sleep duration. For clinicians, identifying children and adolescents who have an unhealthy diet may aid in predicting those at risk of poor mental health. However, future work should strengthen causal claims by employing causal inference designs and investigating potential biological pathways that may explain these relationships.

## Supplementary Information

Supplementary Material 1. Tables A-E are included separately

## Data Availability

Data can be made available upon reasonable request from the first author (CU).

## Abbreviations

ADHD: Attention-deficit/hyperactivity disorder
AMSTAR 2: A Measurement Tool to Assess Systematic Reviews, version 2
CE: Credibility class of evidence
CI: Confidence interval
ESB: Excess significance bias
HR: Hazard ratio
JF: Junk food
k: Number of unique effect estimates in a meta-analysis
LS: Largest-study statistical significance
ns: Non-significant
OR: Odds ratio
PICOS: Population, Intervention/Exposure, Comparison, Outcome, Study design
PRISMA: Preferred Reporting Items for Systematic Reviews and Meta-Analyses
PROSPERO: International Prospective Register of Systematic Reviews
RR: Risk ratio
SSE: Small-study effects (publication bias)
UPF: Ultra-processed food
WDP: Western dietary pattern
WHO: World Health Organization
US: United States
