# Supplementary material for: Association between unhealthy food consumption and mental health outcomes in children and adolescents: an umbrella review
Source: Child Adolesc Psychiatry Ment Health. 2026 Apr 2;20:70. doi: 10.1186/s13034-026-01079-4 (PMC13169651; doi:10.1186/s13034-026-01079-4)
Supplement: Supplementary file 1 — Supplementary Material 1. Tables A-E are included separately [file 13034_2026_1079_MOESM1_ESM.docx]

| **Supplementary Table A.** Preferred Reporting Items for Systematic Reviews and Meta-Analyses (PRISMA) Checklist | | | |
| --- | --- | --- | --- |
| **Section and Topic** | **Item #** | **Checklist item** | **Location where item is reported** |
| **TITLE** | | |  |
| Title | 1 | Identify the report as a systematic review. | Title |
| **ABSTRACT** | | |  |
| Abstract | 2 | See the PRISMA 2020 for Abstracts checklist. | Abstract |
| **INTRODUCTION** | | |  |
| Rationale | 3 | Describe the rationale for the review in the context of existing knowledge. | Intro, paragraphs 1-3 |
| Objectives | 4 | Provide an explicit statement of the objective(s) or question(s) the review addresses. | Intro, last paragraph |
| **METHODS** | | |  |
| Eligibility criteria | 5 | Specify the inclusion and exclusion criteria for the review and how studies were grouped for the syntheses. | Search strategy section, supplementary Table A |
| Information sources | 6 | Specify all databases, registers, websites, organisations, reference lists and other sources searched or consulted to identify studies. Specify the date when each source was last searched or consulted. | Search strategy section |
| Search strategy | 7 | Present the full search strategies for all databases, registers and websites, including any filters and limits used. | Search strategy section, supplementary Table B |
| Selection process | 8 | Specify the methods used to decide whether a study met the inclusion criteria of the review, including how many reviewers screened each record and each report retrieved, whether they worked independently, and if applicable, details of automation tools used in the process. | Screening and selection section |
| Data collection process | 9 | Specify the methods used to collect data from reports, including how many reviewers collected data from each report, whether they worked independently, any processes for obtaining or confirming data from study investigators, and if applicable, details of automation tools used in the process. | Data extraction process section |
| Data items | 10a | List and define all outcomes for which data were sought. Specify whether all results that were compatible with each outcome domain in each study were sought (e.g. for all measures, time points, analyses), and if not, the methods used to decide which results to collect. | Search strategy section, supplementary Table A |
|  | 10b | List and define all other variables for which data were sought (e.g. participant and intervention characteristics, funding sources). Describe any assumptions made about any missing or unclear information. | Data extraction section |
| Study risk of bias assessment | 11 | Specify the methods used to assess risk of bias in the included studies, including details of the tool(s) used, how many reviewers assessed each study and whether they worked independently, and if applicable, details of automation tools used in the process. | Methodological quality assessment section |
| Effect measures | 12 | Specify for each outcome the effect measure(s) (e.g. risk ratio, mean difference) used in the synthesis or presentation of results. | Data extraction section |
| Synthesis methods | 13a | Describe the processes used to decide which studies were eligible for each synthesis (e.g. tabulating the study intervention characteristics and comparing against the planned groups for each synthesis (item #5)). | Screening and selection section |
|  | 13b | Describe any methods required to prepare the data for presentation or synthesis, such as handling of missing summary statistics, or data conversions. | Data Analysis section |
|  | 13c | Describe any methods used to tabulate or visually display results of individual studies and syntheses. | Data extraction section |
|  | 13d | Describe any methods used to synthesize results and provide a rationale for the choice(s). If meta-analysis was performed, describe the model(s), method(s) to identify the presence and extent of statistical heterogeneity, and software package(s) used. | Data Analysis section |
|  | 13e | Describe any methods used to explore possible causes of heterogeneity among study results (e.g. subgroup analysis, meta-regression). | N/A |
|  | 13f | Describe any sensitivity analyses conducted to assess robustness of the synthesized results. | Data Analysis section |
| Reporting bias assessment | 14 | Describe any methods used to assess risk of bias due to missing results in a synthesis (arising from reporting biases). | Methodological quality assessment section |
| Certainty assessment | 15 | Describe any methods used to assess certainty (or confidence) in the body of evidence for an outcome. | Credibility of evidence assessment section |
| **RESULTS** | | |  |
| Study selection | 16a | Describe the results of the search and selection process, from the number of records identified in the search to the number of studies included in the review, ideally using a flow diagram. | Results, first paragraph |
|  | 16b | Cite studies that might appear to meet the inclusion criteria, but which were excluded, and explain why they were excluded. | Figure 1 |
| Study characteristics | 17 | Cite each included study and present its characteristics. | Table 1 |
| Risk of bias in studies | 18 | Present assessments of risk of bias for each included study. | Supplementary Table D |
| Results of in  dividual studies | 19 | For all outcomes, present, for each study: (a) summary statistics for each group (where appropriate) and (b) an effect estimate and its precision (e.g. confidence/credible interval), ideally using structured tables or plots. | Table 1 and Figure 2 |
| Results of syntheses | 20a | For each synthesis, briefly summarise the characteristics and risk of bias among contributing studies. | Methodological quality section |
|  | 20b | Present results of all statistical syntheses conducted. If meta-analysis was done, present for each the summary estimate and its precision (e.g. confidence/credible interval) and measures of statistical heterogeneity. If comparing groups, describe the direction of the effect. | Table 1 |
|  | 20c | Present results of all investigations of possible causes of heterogeneity among study results. | N/A |
|  | 20d | Present results of all sensitivity analyses conducted to assess the robustness of the synthesized results. | Supplementary Figures B and C. |
| Reporting biases | 21 | Present assessments of risk of bias due to missing results (arising from reporting biases) for each synthesis assessed. | Methodological quality section |
| Certainty of evidence | 22 | Present assessments of certainty (or confidence) in the body of evidence for each outcome assessed. | Table 1 and Figure 2 |
| **DISCUSSION** | | |  |
| Discussion | 23a | Provide a general interpretation of the results in the context of other evidence. | Discussion, first paragraph |
|  | 23b | Discuss any limitations of the evidence included in the review. | Strengths and limitations section |
|  | 23c | Discuss any limitations of the review processes used. | Strengths and limitations section |
|  | 23d | Discuss implications of the results for practice, policy, and future research. | Implications and Policy Relevance and Future sections |
| **OTHER INFORMATION** | | |  |
| Registration and protocol | 24a | Provide registration information for the review, including register name and registration number, or state that the review was not registered. | Search strategy section |
|  | 24b | Indicate where the review protocol can be accessed, or state that a protocol was not prepared. | Search strategy section |
|  | 24c | Describe and explain any amendments to information provided at registration or in the protocol. | Search strategy section |
| Support | 25 | Describe sources of financial or non-financial support for the review, and the role of the funders or sponsors in the review. | Funding details section |
| Competing interests | 26 | Declare any competing interests of review authors. | Disclosure Statement section |
| Availability of data, code and other materials | 27 | Report which of the following are publicly available and where they can be found: template data collection forms; data extracted from included studies; data used for all analyses; analytic code; any other materials used in the review. | Data availability section |

*From:*  Page MJ, McKenzie JE, Bossuyt PM, Boutron I, Hoffmann TC, Mulrow CD, et al. The PRISMA 2020 statement: an updated guideline for reporting systematic reviews. BMJ 2021;372:n71. doi: 10.1136/bmj.n71. This work is licensed under CC BY 4.0. To view a copy of this license, visit <https://creativecommons.org/licenses/by/4.0/>

| **Supplementary Table B**. Criteria for defining research question. | |
| --- | --- |
| **PICOS** **Criteria** | **Definition** |
| **Participants** | Included: Studies conducted in children, adolescents, or infants (<19 years)  Excluded: Studies conducted only in adults. Populations not involving humans. |
| **Interventions** | Included: Dietary exposure to five categories of unhealthy food.   1. NOVA-classified UPFs: ultra-processed using culinary preparation techniques and substances not typically found in households. 2. WHO indicators of unhealthy food consumption: foods high in free sugars, saturated fats, salt, and low in nutrient density (e.g., sweets, candies, chocolate, cakes, and cookies). 3. Junk food: foods high in added sugars, fat, and calories with little to no nutritional value and minimal essential nutrients. 4. Fast food: foods typically high in fat, sugar, salt, and calories. 5. Western dietary pattern: typically high in red and processed meats, high-fat dairy products, refined grains, and high-fructose corn syrup.   Excluded: Sugar-sweetened beverages as only exposure. |
| **Comparisons** | Higher intake versus lower intake of unhealthy food. |
| **Outcomes** | Main Outcomes:   1. Physiological health outcomes (e.g., dental caries, asthma, cardiovascular disease) 2. Mental health outcomes (e.g., depression, anxiety, stress) 3. Obesity and body fat parameter outcomes |
| **Study Design** | Included: Systematic reviews with a meta-analysis pooling data focusing on unhealthy food consumption as an exposure.  Excluded: Systematic reviews without meta-analyses. Pooled effect estimates created in a review without a systematic search. |
| Note. WHO=World Health Organization | |

| **Supplementary Table C.** Search Strategy | |
| --- | --- |
| [**Database**](https://pubmed.ncbi.nlm.nih.gov/?term=longquery209fec3f79108877a988) | **Full search terms** |
| [**PubMed**](https://pubmed.ncbi.nlm.nih.gov/?term=longquery209fec3f79108877a988)**/****MEDLINE** | ("ultra-processed food"[Text Word] OR "UPF"[Text Word] OR "processed food"[Text Word] OR "industrial food"[Text Word] OR "unhealthy food"[Text Word] OR "WHO guiding principles"[Title/Abstract:~0] OR "WHO dietary recommendation"[Title/Abstract:~0] OR "Diet, Western"[Mesh] OR "Western Dietary Pattern"[Text Word] OR "WDP"[Text Word] OR "Fast Foods"[Mesh] OR "fast food"[Text Word] OR "junk food"[Text Word] OR "high-sugar food"[Text Word] OR "sugary snacks"[Text Word] OR "high-fat food"[Text Word] OR "high-sodium food"[Text Word] OR "salty snacks"[Text Word] OR "food, processed"[MeSH Terms] OR "Candy"[MeSH Terms:noexp] OR "Candy"[Text Word] OR "candies"[Text Word] OR "sweets"[Text Word])  AND ("Child"[Mesh] OR "Adolescent"[Mesh] OR "Infant"[Mesh] OR "adolescent"[Text Word] OR "child"[Text Word] OR "infant"[Text Word] OR "youth"[Text Word] OR "young people"[Text Word] OR "pediatric"[Text Word] OR "paediatric"[Text Word] OR "children"[Text Word] OR "adolescence"[Text Word] OR "adolescent"[Text Word])  AND ("systematic review"[Title] OR "meta-analysis"[Title] OR "systematic review"[Publication Type] OR "review"[Publication Type] OR "systematic review"[Text Word] OR "systematic reviews as topic"[MeSH Terms]) |
| [**EMBASE**](https://www.embase.com/#advancedSearch/resultspage/history.4/page.1/25.items/orderby.date/source.) | ('ultra-processed food' OR UPF OR 'processed food' OR 'industrial food' OR 'unhealthy food' OR 'WHO guiding principles[Title'/de OR 'WHO dietary recommendation[Title'/de OR 'Diet, Western'/exp OR 'Western Dietary Pattern' OR WDP OR 'Fast Foods'/exp OR 'fast food' OR 'junk food' OR 'high-sugar food' OR 'sugary snacks' OR 'high-fat food' OR 'high-sodium food' OR 'salty snacks' OR 'food, processed'/exp OR Candy/de OR Candy OR candies OR sweets)  AND (Child/exp OR Adolescent/exp OR Infant/exp OR adolescent OR child OR infant OR youth OR 'young people' OR pediatric OR paediatric OR children OR adolescence OR adolescent)  AND ('systematic review':ti OR meta-analysis:ti OR term:it OR term:it OR 'systematic review' OR 'systematic reviews as topic'/exp) |
| [**Scopus**](https://www.scopus.com/results/results.uri?sort=plf-f&src=s&sid=cb35b38dfc1332d8a973091395444bca&sot=a&sdt=a&sessionSearchId=cb35b38dfc1332d8a973091395444bca&origin=searchadvanced&editSaveSearch=&txGid=0a98149c0d34ca4d605c3d31f489425c&limit=10&s=%28TITLE-ABS-KEY%28%22ultra-processed+food%22%29+OR+TITLE-ABS-KEY%28UPF%29+OR+TITLE-ABS-KEY%28%22processed+food%22%29+OR+TITLE-ABS-KEY%28%22industrial+food%22%29+OR+TITLE-ABS-KEY%28%22unhealthy+food%22%29+OR+INDEXTERMS%28%22WHO+guiding+principles%5BTitle%22%29+OR+INDEXTERMS%28%22WHO+dietary+recommendation%5BTitle%22%29+OR+INDEXTERMS%28%22Diet%2C+Western%22%29+OR+TITLE-ABS-KEY%28%22Western+Dietary+Pattern%22%29+OR+TITLE-ABS-KEY%28WDP%29+OR+INDEXTERMS%28%22Fast+Foods%22%29+OR+TITLE-ABS-KEY%28%22fast+food%22%29+OR+TITLE-ABS-KEY%28%22junk+food%22%29+OR+TITLE-ABS-KEY%28%22high-sugar+food%22%29+OR+TITLE-ABS-KEY%28%22sugary+snacks%22%29+OR+TITLE-ABS-KEY%28%22high-fat+food%22%29+OR+TITLE-ABS-KEY%28%22high-sodium+food%22%29+OR+TITLE-ABS-KEY%28%22salty+snacks%22%29+OR+INDEXTERMS%28%22food%2C+processed%22%29+OR+INDEXTERMS%28Candy%29+OR+TITLE-ABS-KEY%28Candy%29+OR+TITLE-ABS-KEY%28candies%29+OR+TITLE-ABS-KEY%28sweets%29%29+AND+%28INDEXTERMS%28Child%29+OR+INDEXTERMS%28Adolescent%29+OR+INDEXTERMS%28Infant%29+OR+TITLE-ABS-KEY%28adolescent%29+OR+TITLE-ABS-KEY%28child%29+OR+TITLE-ABS-KEY%28infant%29+OR+TITLE-ABS-KEY%28youth%29+OR+TITLE-ABS-KEY%28%22young+people%22%29+OR+TITLE-ABS-KEY%28pediatric%29+OR+TITLE-ABS-KEY%28paediatric%29+OR+TITLE-ABS-KEY%28children%29+OR+TITLE-ABS-KEY%28adolescence%29+OR+TITLE-ABS-KEY%28adolescent%29%29+AND+%28TITLE%28%22systematic+review%22%29+OR+TITLE%28meta-analysis%29+OR+DOCTYPE%28%22systematic+review%22%29+OR+DOCTYPE%28review%29+OR+TITLE-ABS-KEY%28%22systematic+review%22%29+OR+INDEXTERMS%28%22systematic+reviews+as+topic%22%29%29) | (TITLE-ABS-KEY("ultra-processed food") OR TITLE-ABS-KEY(UPF) OR TITLE-ABS-KEY("processed food") OR TITLE-ABS-KEY("industrial food") OR TITLE-ABS-KEY("unhealthy food") OR INDEXTERMS("WHO guiding principles[Title") OR INDEXTERMS("WHO dietary recommendation[Title") OR INDEXTERMS("Diet, Western") OR TITLE-ABS-KEY("Western Dietary Pattern") OR TITLE-ABS-KEY(WDP) OR INDEXTERMS("Fast Foods") OR TITLE-ABS-KEY("fast food") OR TITLE-ABS-KEY("junk food") OR TITLE-ABS-KEY("high-sugar food") OR TITLE-ABS-KEY("sugary snacks") OR TITLE-ABS-KEY("high-fat food") OR TITLE-ABS-KEY("high-sodium food") OR TITLE-ABS-KEY("salty snacks") OR INDEXTERMS("food, processed") OR INDEXTERMS(Candy) OR TITLE-ABS-KEY(Candy) OR TITLE-ABS-KEY(candies) OR TITLE-ABS-KEY(sweets))  AND (INDEXTERMS(Child) OR INDEXTERMS(Adolescent) OR INDEXTERMS(Infant) OR TITLE-ABS-KEY(adolescent) OR TITLE-ABS-KEY(child) OR TITLE-ABS-KEY(infant) OR TITLE-ABS-KEY(youth) OR TITLE-ABS-KEY("young people") OR TITLE-ABS-KEY(pediatric) OR TITLE-ABS-KEY(paediatric) OR TITLE-ABS-KEY(children) OR TITLE-ABS-KEY(adolescence) OR TITLE-ABS-KEY(adolescent))  AND (TITLE("systematic review") OR TITLE(meta-analysis) OR DOCTYPE("systematic review") OR DOCTYPE(review) OR TITLE-ABS-KEY("systematic review") OR INDEXTERMS("systematic reviews as topic")) |
| [**CINAHL**](https://web.p.ebscohost.com/ehost/resultsadvanced?vid=40&sid=e3ed0d26-3310-4877-a197-e89cc7a82ed8%40redis) | ("ultra-processed food" OR UPF OR "processed food" OR "industrial food" OR "unhealthy food" OR (MH "WHO guiding principles[Title") OR (MH "WHO dietary recommendation[Title") OR (MH "Diet, Western+") OR "Western Dietary Pattern" OR WDP OR (MH "Fast Foods+") OR "fast food" OR "junk food" OR "high-sugar food" OR "sugary snacks" OR "high-fat food" OR "high-sodium food" OR "salty snacks" OR (MH "food, processed+") OR (MH Candy) OR Candy OR candies OR sweets)  AND ((MH Child+) OR (MH Adolescent+) OR (MH Infant+) OR adolescent OR child OR infant OR youth OR "young people" OR pediatric OR paediatric OR children OR adolescence OR adolescent)  AND ((TI "systematic review") OR (TI meta-analysis) OR (PT "systematic review") OR (PT review) OR "systematic review" OR (MH "systematic reviews as topic+")) |

| **Supplementary Table D.** Credibility of Evidence Assessment | |
| --- | --- |
| **Assessment Class** | **Criteria** |
| I – Convincing Class | - n of cases >1,000 - *P* < 10^-6^ - I^2^ < 50% - No small-study effects - No excess significance bias - Largest study statistically significant |
| II – Highly Suggestive Class | - Above criteria not met - n of cases >1,000 - *P* < 10^-6^ - Largest study statistically significant |
| III – Suggestive Class | - Above criteria not met - n of cases >1,000 - *P* < 10^-3^ |
| IV – Weak Class | - Above criteria not met - *P* < 0.05 |
| ns – Nonsignificant Class | - *P* > 0.05 |

| **Supplementary Table E**: Methodological quality assessment of meta-analyses using AMSTAR 2 | | | | | | | | | | | | | | | | | | | |
| --- | --- | --- | --- | --- | --- | --- | --- | --- | --- | --- | --- | --- | --- | --- | --- | --- | --- | --- | --- |
| Study ID | AMSTAR 2 Checklist | | | | | | | | | | | | | | | | |  |  |
|  | Q1 | Q2 | Q3 | Q4 | Q5 | Q6 | Q7 | Q8 | Q9 | Q10 | Q11 | Q12 | Q13 | Q14 | Q15 | Q16 | Rating | |  |
| Delpino 2023 | Yes | Yes | Yes | Yes | Yes | Yes | Yes | Yes | Yes | Partial | Yes | Yes | No | Partial | Partial | No | Low | |  |
| Malmir 2023 | Yes | No | Yes | Yes | Yes | Yes | Yes | Yes | Yes | No | Yes | Yes | No | Yes | Partial | No | Critically low | |  |
| Lane 2022 | Yes | Yes | Yes | Yes | Yes | Yes | Yes | Yes | Yes | No | Yes | Yes | Yes | Yes | No | Yes | Low | |  |
| Shareghfarid 2020 | Yes | No | Yes | Yes | Yes | Yes | Yes | Yes | Yes | No | Yes | Yes | Yes | Yes | No | No | Critically low | |  |
| Pourmotabbed 2024 | Yes | Yes | Yes | Yes | Yes | Yes | Yes | Yes | Yes | No | Yes | Yes | Yes | Yes | Yes | No | High | |  |
| Khazdouz 2024 | Yes | Yes | Yes | Yes | Yes | Yes | Partial | Yes | Yes | No | Yes | Yes | Yes | Yes | Yes | No | Moderate | |  |
| Zhong 2024 | Yes | No | Yes | Yes | Yes | Yes | No | Yes | Yes | No | Yes | Yes | Partial | Yes | No | No | Critically low | |  |
| Note. AMSTAR 2 critical domain columns are shaded grey, and corresponding critical questions are bolded below.  Q1: Did the research questions and inclusion criteria for the review include the components of PICO? **Q2: Did the report of the review contain an explicit statement that the review methods were established prior to the conduct of the review and did the report justify any significant deviations from the protocol?** Q3: Did the review authors explain their selection of the study designs for inclusion in the review? **Q4: Did the review authors use a comprehensive literature search strategy?** Q5: Did the review authors perform study selection in duplicate? Q6: Did the review authors perform data extraction in duplicate? **Q7: Did the review authors provide a list of excluded studies and justify the exclusions?** Q8: Did the review authors describe the included studies in adequate detail? **Q9: Did the review authors use a satisfactory technique for assessing the risk of bias (RoB) in individual studies that were included in the review?**  Q10: Did the review authors report on the sources of funding for the studies included in the review? **Q11: If meta-analysis was performed, did the review authors use appropriate methods for statistical combination of results?** Q12: If meta-analysis was performed, did the review authors assess the potential impact of RoB in individual studies on the results of the meta-analysis or other evidence synthesis? **Q13: Did the review authors account for RoB in primary studies when interpreting/discussing the results of the review?** Q14: Did the review authors provide a satisfactory explanation for, and discussion of, any heterogeneity observed in the results of the review? **Q15: If they performed quantitative synthesis did the review authors carry out an adequate investigation of publication bias (small study bias) and discuss its likely impact on the results of the review?** Q16: Did the review authors report any potential sources of conflict of interest, including any funding they received for conducting the review? | | | | | | | | | | | | | | | | | | | |

**Supplementary Figure A**. Overlap between effect estimates used in meta-analyses.
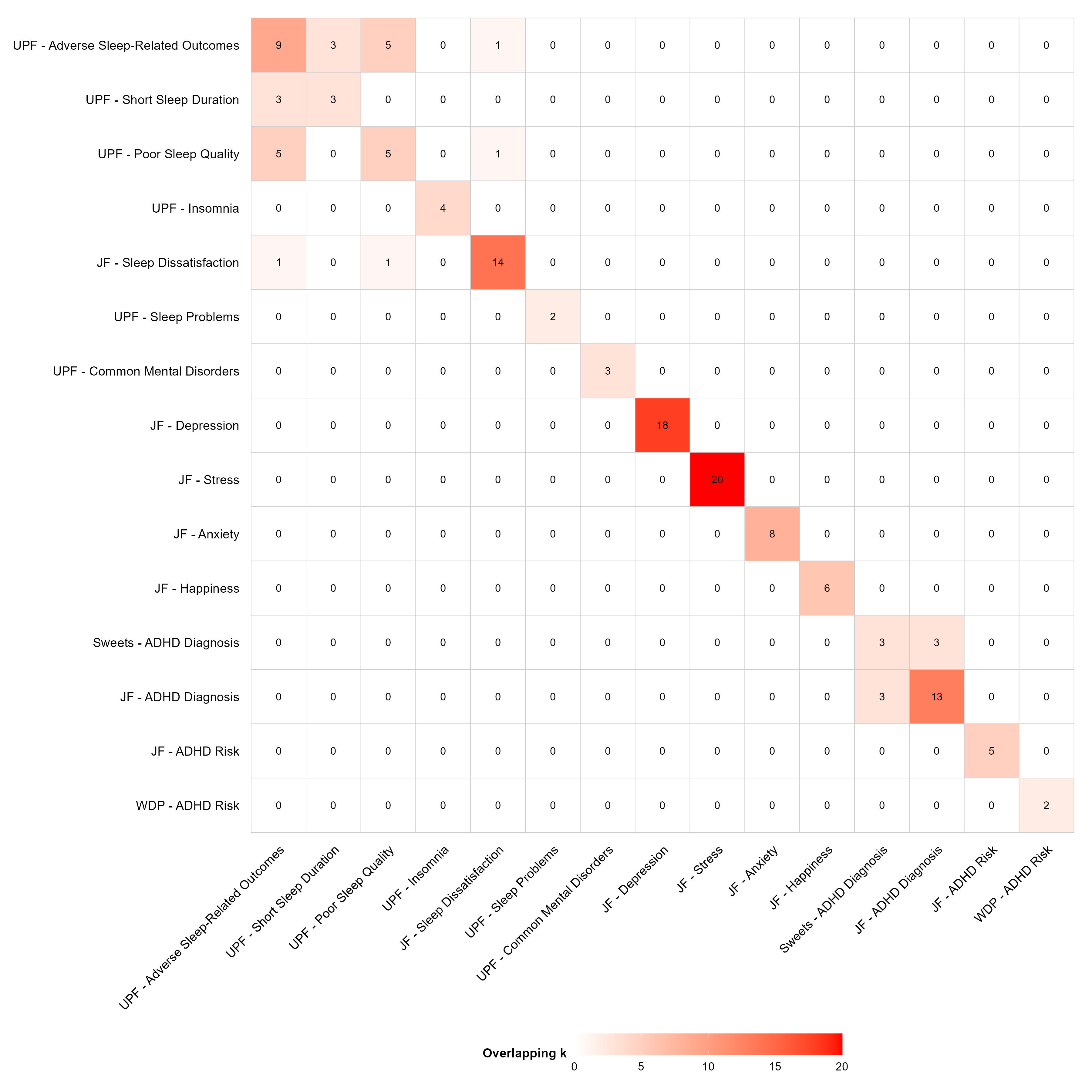


**Note**. k=number of unique effect estimates included in each meta-analysis, UPF = ultra-processed foods, JF = junk food.

**Supplementary Figure B.** Sensitivity analysis to exclude study effect overlap.


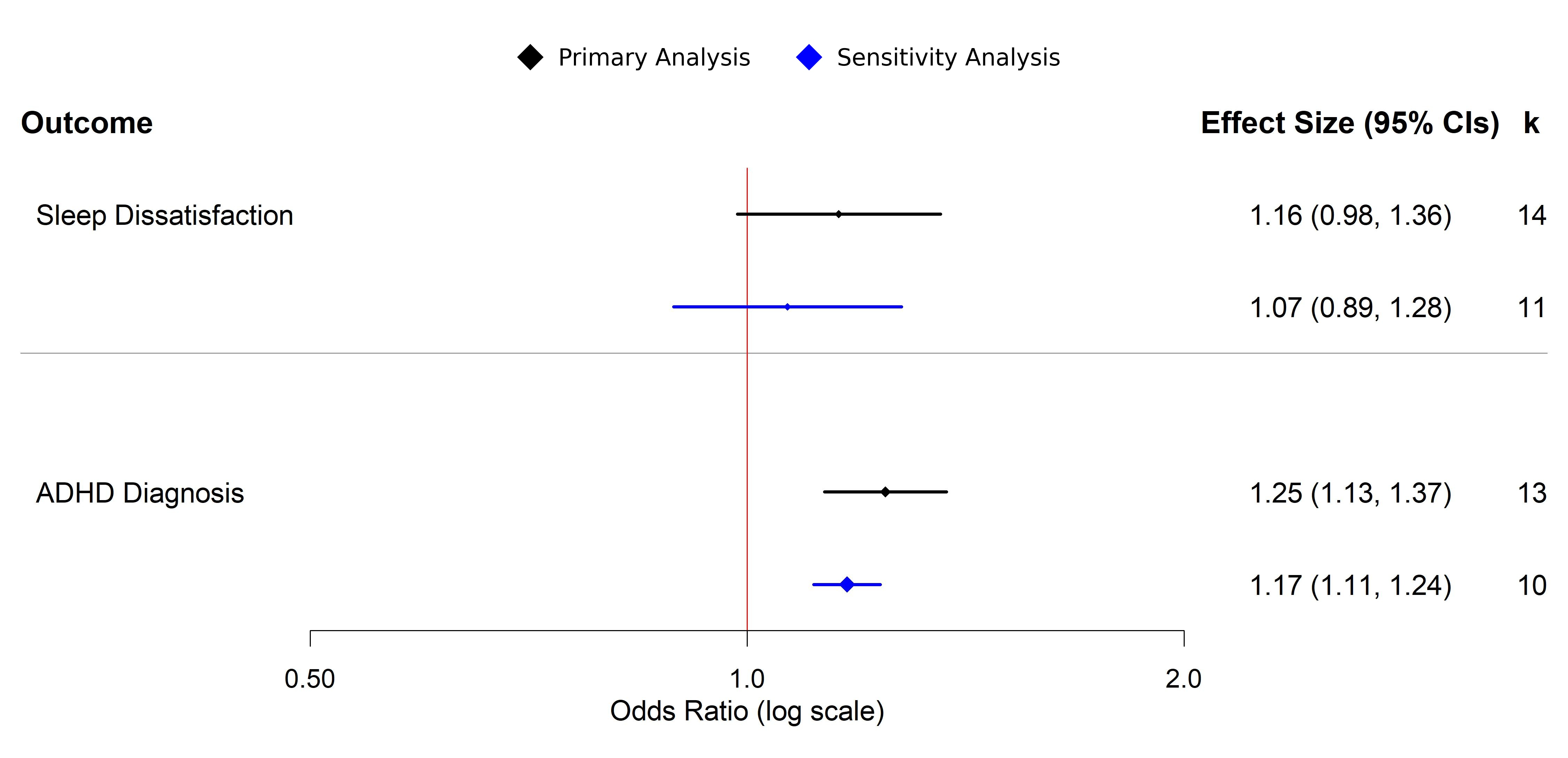

Note. Exposures are junk food for both outcomes. K=number of unique effect estimates included in each meta-analysis.

**Supplementary Figure C.** Sensitivity analysis to control heterogeneity (I2) to below 50% using sequential exclusion algorithm.
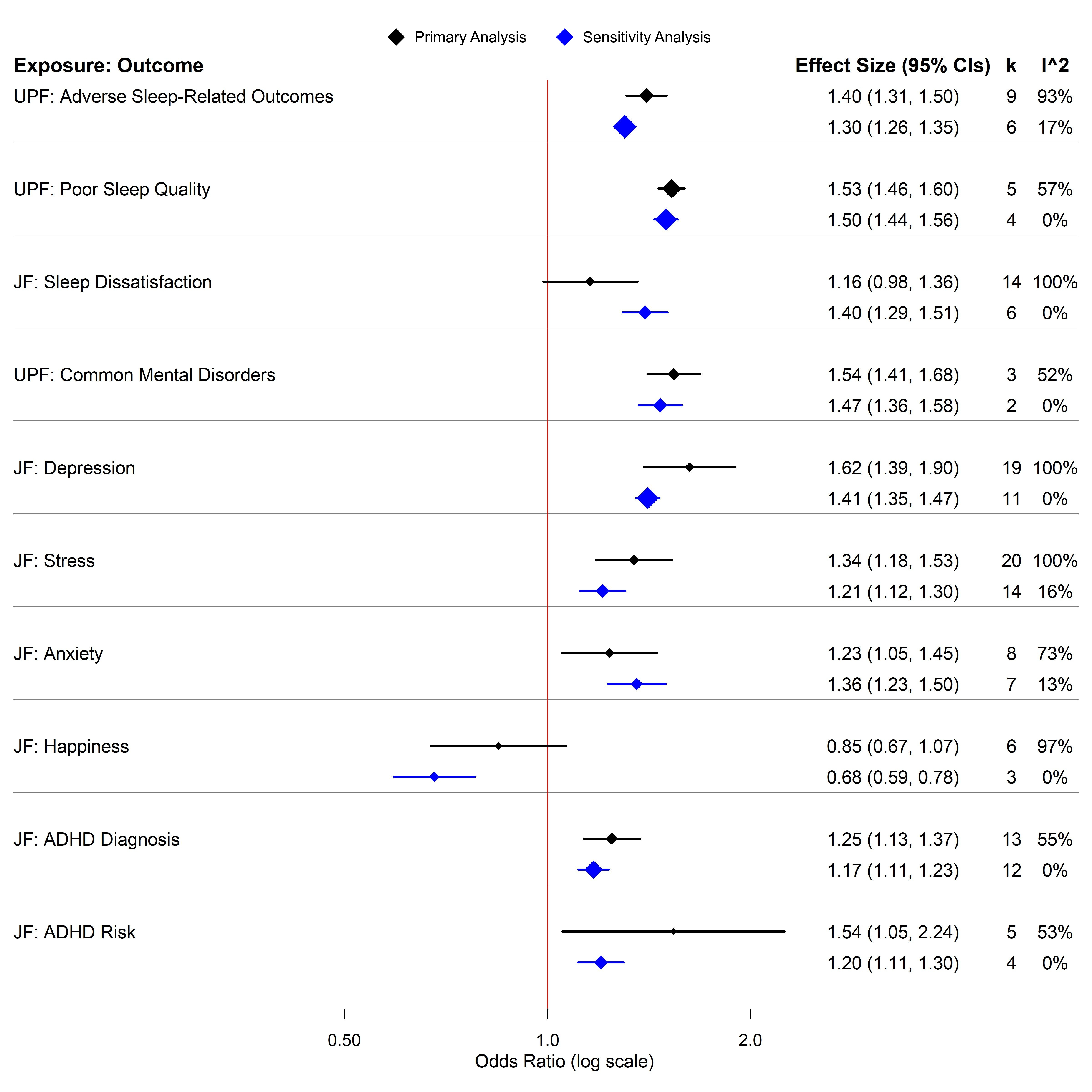


**Note.** K=number of unique effect estimates included in each meta-analysis, UPF = ultra-processed foods, JF = junk food, I^2 = I^2^ statistic.

Screening conception to December 2023

- PubMed/MEDLINE, Scopus, Embase, and CINAHL.
- Original search covered inception to December 2023; will be updated to December 2024.
- Initial search: 1,567 articles.
- After removing 561 duplicates, 1,006 references remained.
- 62 full texts reviewed; 14 systematic reviews with meta-analyses were ultimately included.

Updated search from December 2023 to January 2025

- PubMed/MEDLINE, Scopus, Embase, and CINAHL.
